# The impact of data protection laws on Chinese digital cross-border mergers and acquisitions: A quasi-natural experiment based on the EU’s general data protection regulation

**DOI:** 10.1371/journal.pone.0331246

**Published:** 2026-01-02

**Authors:** Miaozhi Yu, Xiaoshuang Ye

**Affiliations:** 1 School of Economics, Zhejiang University of Technology, Hangzhou, China; 2 Institute for Industrial System Modernization, Zhejiang University of Technology, Hangzhou, China; University of Science and Technology of China, CHINA

## Abstract

With the rapid growth of the digital economy, international investment activities exemplified by digital cross-border mergers and acquisitions (M&A) are increasingly active. Does the host country’s data regulation affect China’s digital cross-border M&A? This study, based on a panel dataset of Chinese digital cross-border M&A from 2009 to 2021, evaluates the impact of the European Union’s General Data Protection Regulation (GDPR) on China’s digital cross-border M&A using the PSM-DID model. The research findings indicate: (1) The implementation of GDPR significantly adversely affects Chinese enterprises engaging in digital cross-border M&A within the European Union (EU), a conclusion upheld after various robustness checks; (2) Heterogeneity analysis reveals that compared to lower-middle-income countries, high-income countries’ GDPR policies exert a more pronounced inhibiting effect on China’s digital cross-border M&A; (3) Moderation effect analysis suggests that the improvement of the digital infrastructure level, the expansion of market size, and reduction in trade costs within EU countries will mitigate GDPR’s inhibitory effect on Chinese enterprises conducting digital cross-border M&A.

## 1. Introduction

In the contemporary era, digital technology and the digital economy epitomize the vanguard of the global technological revolution and industrial metamorphosis, constituting pivotal focal points in the current landscape of international competition. Nations across the globe are intensifying efforts to propel the development of critical sectors within the digital economy, which has garnered considerable attention for its notable strides in advancement. As per the “Global Digital Economy White Paper (2023)” released by the China Academy of Information and Communications Technology in July 2023, the collective digital economy of five major nations—United States, China, Germany, Japan, and South Korea—amounted to $31 trillion in 2022, registering a 7.6% year-on-year growth, surpassing the GDP growth rate by 5.4 percentage points. Simultaneously, owing to escalating global market demands for digital products and services, digital cross-border M&A, with their inherent advantages of low marginal costs, substantial economies of scale, broad reach, and network effects, have emerged as hotspots for global cross-border investments, injecting fresh impetus into global investment growth. Safeguarding data security stands as the cornerstone and prerequisite for advancing the digital economy. However, the rapid evolution of data-centric digital cross-border M&A has also precipitated numerous data-related issues such as monopolies by platform corporations, big data-enabled price discrimination against existing customers, the proliferation of online black market, and breaches of privacy. To address these concerns, countries worldwide have instituted legislation and standards focused on data security and the protection of personal information. Notably, the “General Data Protection Regulation” (GDPR) enacted by the EU in May 2018 is hailed as the most stringent data protection law, aiming to regulate and constrain corporate handling and circulation of personal data to safeguard individual data rights. Data, utilized extensively as a production factor, harbors both positive and negative effects. Hence, the implementation of data protection laws raises a fundamental question regarding its impact on digital cross-border M&A—whether positive or negative—an issue demanding urgent attention and scholarly exploration in today’s academic realm.

Based on existing literature [1, 2], digital cross-border M&A generally refer to cross-border acquisitions conducted by firms to enhance their digital capabilities. Academics typically categorize digital cross-border M&A into the following three types based on the purpose of the acquisition [3]: (1) Technology acquisition: Acquiring strategic digital technologies such as cybersecurity, artificial intelligence, or cloud technologies to promote innovation in the acquiring firm’s digital technology; (2) Service acquisition: Acquiring companies in fields such as data analytics, software services, or digital financial services to improve the acquirer’s digital capabilities and service levels; (3) Market acquisition: Acquiring platform-based companies to gain digital marketing capabilities, expand online sales channels, and optimize consumer-facing supply chains to capture market share. According to institutional theory, the institutional environment directly affects transaction costs, which in turn determine the risks and returns of economic activities [4]. The institutional environment can be divided into formal institutions (such as political, economic, and legal systems) and informal institutions (such as customs, norms, and culture). In the early stages of digital economic activity, formal institutions have a particularly significant impact on digital trade and cross-border M&A [5]. Therefore, this paper explores the drivers of digital M&A from the perspective of formal institutions. Currently, the innovation and iteration of digital technologies are primarily led by developed countries, which place greater emphasis on digital technology protection and data privacy compared to emerging economies [6]. For instance, the United States and the EU have successively introduced a series of digital technology and data protection policies, establishing corresponding regulatory frameworks. However, China’s digital M&A market is still in a developmental stage and remains relatively immature compared to developed economies such as those in Europe and North America. Many Chinese enterprises face numerous challenges during the valuation and negotiation processes of cross-border M&A. In particular, with the rise of nationalism and the increasingly prominent trend of de-globalization in the international economic environment, countries have been tightening their scrutiny of foreign acquisitions, especially in the digital and technology sectors, making it more difficult for firms to complete cross-border digital M&A [7]. Therefore, this paper takes the EU’s GDPR as a case study to explore the impact of data protection laws on Chinese digital cross-border M&A activities. It reveals how stringent data protection regulations influence M&A strategies during cross-border digital acquisitions, thus enriching existing research in this field.

This study makes two significant contributions. First, it innovatively integrates the EU’s GDPR policy into the analytical framework, broadening the range of factors influencing cross-border M&A. By combining the digital economy with cross-border M&A, the paper examines the impact of GDPR on digital cross-border M&A, not only extending the research boundaries of cross-border M&A in the digital age but also offering new insights for governments on how to guide digital enterprises to ‘go global’ under GDPR. Second, this study delves into the mechanisms through which GDPR affects Chinese digital cross-border M&A, providing a novel evaluation of its moderating effects from the perspectives of digital infrastructure, market size, and trade costs. It explores research dimensions that have previously been overlooked, making a noteworthy contribution to our understanding of the policy effects of data protection laws.

The remainder of this paper is structured as follows: Section 2 provides a literature review. Section 3 explains the institutional background and theoretical framework. Section 4 describes the model setup and data. Section 5 presents the empirical analysis in detail. Section 6 examines the mechanisms. Finally, the last section summarizes the empirical findings and offers appropriate policy recommendations.

## 2. Literature review

Presently, research on cross-border M&A primarily focuses on the influencing factors and performance, emphasizing the understanding of their decision-making basis and processes. Studies on the influencing factors of cross-border M&A mainly concentrate on aspects such as market size [8], geographical and cultural distances [9, 10], financial markets [11], trade barriers [12], institutional quality [13], industrial policies [14], among others. In recent years, with the rapid development of digital technology and the growing market demand for innovative digital products, there has been a surge in digital cross-border M&A globally, attracting widespread attention in academic circles. Jiang and Tang, from the perspective of characteristics and driving factors, found that the host country’s core technological capabilities and research and development resources serve as the primary driving forces for digital cross-border M&A, while geographical distance and the host country’s market size are no longer significant factors influencing enterprise cross-border M&A. Wang et al. [15] found that the level of development of the digital economy is a key factor in attracting digital cross-border M&A, and digital distance may hinder cross-border M&A between countries.

As the digital economy continues to advance, new digital regulations continue to emerge. Scholars have begun studying the policy effects of personal data protection. Sabatino and Sapi [16] used retail companies operating in North America or the European Union as a sample. They employed a triple difference-in-differences (DDD) model to explore the impact of the 2009 revision of the EU’s Privacy and Electronic Communications Directive (ePrivacy) on the structure of the e-commerce market. The study found that the 2009 ePrivacy revision had differential effects on the revenue of e-commerce companies of various sizes. Notably, the revenue of large firms significantly decreased, while small firms experienced no significant impact. Patel et al. [17] studied the economic effects of the California Consumer Privacy Act (CCPA) and found no significant stock market reactions within the sample of retail companies. The formal enactment of the EU’s GDPR marked a significant shift in the landscape of data protection [18]. The general principles of GDPR require explicit consent for data processing, data minimization, and grant users the right to access and erase their data [19]. With its strict compliance requirements and heavy fines, GDPR has become the ‘gold standard’ for data protection laws globally [20], and similar laws have been adopted or proposed in various regions. Due to the adoption and enforcement of GDPR, some scholars have begun to study its economic and societal impacts. Farhad [21] examined 200 companies across North America, Europe, Asia, and Africa, employing a mixed methods research (MMR) approach to investigate the impact of data protection laws on the business models of technology companies. The study found that while the implementation of GDPR posed operational challenges for sub-sectors such as fintech and healthtech, increasing compliance costs, it also, to some extent, drove technological innovation within companies. Other scholars have conducted empirical studies from the perspective of firm performance, finding that GDPR’s implementation harmed the profitability of companies targeting European consumers through cost-driven strategies. Congiu et al. [22], whose research is closely related to this study, used websites from U.S. states as a control group and employed a difference-in-differences (DID) model to evaluate the impact of GDPR on website traffic and user engagement. Their findings showed that following the implementation of GDPR, website engagement significantly decreased, and the relationship between website size and traffic changes due to GDPR followed an inverted U-shape. Another study also used the EU’s GDPR as a quasi-natural experiment to explore the effect of data protection laws on Chinese e-commerce cross-border M&A. Ma et al. 10 found that the implementation of GDPR significantly suppressed Chinese e-commerce cross-border M&A, with a notable anticipation effect. This paper reviews the above-related literature, as shown in Table 1.

**Table 1:** Research Status.

	Research Perspective	Research Content	Representative Authors
M&A	Influencing factors	Mainly focusing on market size, geographic and cultural distance, financial markets, trade barriers, and institutional quality.	Ahmad and Lambert(2018); Li and Yang(2020); Erel et al. (2012); Ma et al. (2023); Breinlich(2008); Pandey et al.(2023)
	Digital cross-border M&A	Core technologies and R&D resources are the primary drivers of digital cross-border M&A, while the level of development of the digital economy is a key factor in attracting such M&A.	Jiang and Tang(2021); Wang et al.(2024)
Data protection laws	GDPR	It has a certain impact on company business models, performance, website traffic, user engagement, and cross-border e-commerce M&A.	Farhad (2024); Frey and Presidente(2024); Congiu et al.(2022); Ma et al.(2023)
	Other data protection regulations	ePrivacy has a significant impact on the structure of the e-commerce market, while CCPA did not produce a significant stock market reaction in the sample of retail companies.	Sabatino and Sapi(2022); Patel et al.(2023)

In summary, the existing literature has the following shortcomings: (1) From a research perspective, most studies on cross-border M&A focus on factors such as market size and distance, with little consideration given to the impact of data regulation policies. (2) In terms of research content, studies on the economic and social impacts of GDPR are primarily centered on European and American countries, and are limited to analyses of the e-commerce industry, business models, and corporate performance, without addressing the cross-border operations of digital enterprises. (3) Regarding research methods, there has been a lack of in-depth analysis of the theoretical mechanisms through which data regulations produce economic effects. Based on these gaps, this paper presents the following innovations: (1) Innovation in research perspective. By taking GDPR as a case, this study analyzes the impact of host country data protection on China’s digital cross-border M&A, expanding the research boundaries of cross-border M&A in the digital economy era. (2) Innovation in research content. This paper focuses on China’s digital cross-border M&A activities, placing particular emphasis on international economic activities that are more susceptible to data regulations, thereby providing a valuable supplement to existing studies. (3) Innovation in research methods. This study examines the impact of GDPR on China’s digital cross-border M&A from a cost-benefit perspective, and uniquely evaluates its moderating effects from the aspects of digital infrastructure, market size, and trade costs. This approach contributes to a more comprehensive understanding of how data protection policies influence real economic activities.

## 3. Institutional background and theoretical analysis

### 3.1. Institutional background

As the global digital economy continues to evolve, understanding and studying data protection laws and their impact has become increasingly crucial. This issue not only encompasses data sovereignty and privacy protection but also relates to the future of economic growth, particularly in the context of digital cross-border M&A. According to the regulations established by various countries, governance frameworks can be broadly classified into three categories: market-oriented, represented by the United States; externally strict regulatory, represented by the European Union; and national security-focused, represented by China [23, 24]. Table 2 presents the current state of data protection governance in these three major economies.

**Table 2:** Current State of Data Protection Governance in Representative Economies.

Economies	Stances	Practices
United States	Advocate for the free flow of data and rely on market mechanisms to protect privacy.	The U.S. adopts a market-oriented approach to regulating cross-border data flows, emphasizing commercial data and prioritizing the protection of private enterprise interests. Unlike the EU, the U.S. lacks a unified enforcement body comparable to the EU Data Protection Supervisory Authority (EDPS). Instead, it relies on industry self-regulation to address privacy protection issues in cross-border data flows.
EU	Advocate for strict control of personal data, emphasizing the protection of individual consumer interests.	The EU, grounded in fundamental human rights, regards the governance of data and its cross-border flow as a critical component of national sovereignty. To this end, it has established a series of rules to manage and regulate these processes. In 2016, the EU introduced the GDPR, which took effect in 2018. The GDPR establishes mandatory rules for processing personal data and imposes stringent restrictions on cross-border data transfers.
China	Advocate for a regulatory framework that implements data localization guided by ‘sovereignty protection.’	The core of China’s data protection laws is data security, which fundamentally prioritizes national security interests. Through a series of laws, regulations, and rules related to data export, such as the Cybersecurity Law, Data Security Law, and Personal Information Protection Law, China has established a basic management model of ‘localized storage plus security assessment exceptions’ for important data and personal data.

Since its implementation, the EU’s GDPR has become the “gold standard” for global data protection laws due to its stringent compliance requirements and substantial penalty mechanisms. The GDPR not only imposes clear restrictions on the use of personal data and its cross-border flow but also extends its influence beyond the geographical boundaries of the EU through its principle of “extraterritoriality.” This means that even if a company is not located within the EU, it must still comply with the GDPR as long as it processes data of EU residents. Therefore, studying the impact of the EU’s GDPR can more effectively illustrate how stringent data protection laws affect cross-border M&A activities.

In contrast, data protection laws in the United States are relatively lenient, emphasizing industry self-regulation and market mechanisms. This results in a lighter regulatory burden for companies engaging in cross-border M&A. Meanwhile, China has reinforced data localization requirements and restrictions on data exportation through laws such as the Cybersecurity Law, highlighting the primacy of data sovereignty and national security, which leads to higher compliance costs. In these varying data protection environments, multinational enterprises may engage in “regulatory arbitrage,” opting to conduct M&A activities in countries with more relaxed data protection requirements to reduce compliance costs and circumvent stringent regulations.

### 3.2. Theoretical analysis and research hypotheses

According to the principle of maximizing corporate profits, the decision of whether a company engages in cross-border M&A is primarily influenced by two factors: costs and benefits. Over the long term, if the costs incurred are lower than the discounted expected returns, it is suitable to engage in cross-border M&A; otherwise, it is not suitable11. Therefore, concerning the impact of GDPR on digital cross-border M&A, this study attempts to analyze it from the perspectives of costs and benefits.

(1) Regarding costs: Firstly, for digital enterprises, the accessibility of data and the availability of digital technology are crucial prerequisites for engaging in cross-border M&A. However, the implementation of GDPR might raise the market entry barrier for non-EU digital enterprises, subjecting them to stricter regulations, potentially increasing government-set penalties [25]. Consequently, when non-EU enterprises conduct digital cross-border M&A within the EU, obtaining data and digital technology might become challenging, leading to a substantial increase in information costs. Secondly, non-EU digital enterprises, to comply with GDPR, need to augment activities such as evaluating compliance deficiencies, employee training, continuous supervision for compliance, and consulting external compliance experts, incurring high costs for data compliance [26]. Lastly, compared to non-EU digital enterprises, EU digital enterprises have not faced GDPR restrictions and, instead, seem to receive certain preferential treatment. GDPR has altered the past competitive landscape in the EU market, reducing the market competitiveness of non-EU digital enterprises. The uncertainty and compliance risks associated with non-EU enterprises conducting digital cross-border M&A within the EU have significantly increased, resulting in certain risk costs.
(2) In terms of benefits: The implementation of GDPR might restrict the capability of non-EU digital enterprises to collect, process, and update consumer personal data, leading to issues like information asymmetry and inaccurate information management. These issues might prevent non-EU digital enterprises from better meeting personalized consumer goods demands, hindering the matching of supply and demand relationships between buyers and sellers, consequently weakening the market competitiveness of non-EU digital enterprises. This decline could cause a significant decrease in transaction volume and reduced profits for non-EU digital enterprises [27], restraining the willingness of non-EU enterprises for digital cross-border M&A. Therefore, from the perspectives of costs and benefits, the implementation of GDPR is likely to inhibit Chinese enterprises from conducting digital cross-border M&A within the EU. Based on this analysis, this paper proposes the first hypothesis:

Hypothesis 1: The implementation of GDPR inhibits Chinese enterprises from engaging in digital cross-border M&A within the EU.

In comparison to traditional cross-border M&A, digital cross-border M&A presents distinct features such as enhanced information transparency, significant economies of scale3, and lower marginal costs [28]. These traits potentially determine the specific pathway through which GDPR affects digital cross-border M&A. Subsequently, building upon the preceding sections, this paper proceeds to theoretically analyze, from the perspectives of digital infrastructure, market scale, and trade costs, the moderating effects on the relationship between GDPR and digital cross-border M&A.

Firstly, examining the mechanism of advancing digital infrastructure development: (1) The construction and evolution of digital infrastructure, exemplified by the internet, transcend temporal and spatial constraints on information dissemination, accelerating the pace and expanding the scope of information transmission [29], facilitating digital enterprises’ access to information and data. (2) The continual elevation of digital infrastructure development, including the internet, aids in disrupting traditional economic development patterns, deepening the integration of information and communication technology with industries [30], mitigating information asymmetry issues, enhancing resource allocation efficiency, thereby fostering the formation of economies of scale. (3) The advancement in digital infrastructure levels benefits digital enterprises in utilizing internet technology to mine consumer behavior big data, guiding novel consumer demands, thereby amplifying market requirements. Consequently, the elevation of the host country’s digital infrastructure level contributes to attracting foreign enterprises to engage in digital cross-border M&A, thus weakening the inhibitory effects of GDPR. Based on these analyses, this paper proposes the second hypothesis:

Hypothesis 2: As the level of digital infrastructure construction increases, the effect of GDPR restricting Chinese enterprises from conducting digital cross-border M&A in the EU gradually diminishes.

Next, examining the mechanism of expanding market scale. Expanding overseas markets constitutes a primary motivation for enterprises engaging in foreign direct investment [31], and cross-border M&A represent a crucial approach for companies involved in foreign direct investment (FDI). (1) The expansion of a country’s market scale implies an increase in market capacity and consumer potential [32]. As the market scale of the host country enlarges, the likelihood of benefits for foreign enterprises in that country continually rises, thereby augmenting the attractiveness of the country to foreign enterprises, encouraging companies to initiate cross-border M&A in that country. (2) With the rise of the digital economy, consumers’ traditional subjective judgments are gradually evolving, enabling consumers to search for their preferred goods through digital tools like search engines, leading to a growing demand for differentiated and personalized products. When a country’s market scale expands, digital enterprises can efficiently aggregate and integrate consumers’ diversified needs via platforms, allowing for more targeted personalized recommendations, enhancing the liquidity of the “long tail market,” consequently generating economies of scale at lower costs, thus attracting foreign enterprises to engage in digital cross-border M&A. Consequently, the expansion of the host country’s market scale contributes to attracting foreign enterprises to conduct digital cross-border M&A, thereby mitigating the inhibitory effects of GDPR. Based on this, this paper proposes the third hypothesis:

Hypothesis 3: As the market scale expands, the restraining effect of GDPR on Chinese enterprises conducting digital cross-border M&A in the EU gradually diminishes.

Finally, examining the mechanism of increased trade costs. Presently, within the academic domain, there are two primary perspectives regarding the relationship between trade costs and FDI: (1) Trade costs exhibit a positive correlation with FDI. The proximity-concentration trade-off theory and the “tariff-jumping” hypothesis support this viewpoint, suggesting that when a country’s trade costs are higher, multinational corporations reduce their exports to that country and instead opt for overseas investments, thereby promoting corporate investment. Many scholars have empirically examined and validated this perspective [33,34]; (2) Trade costs show a negative correlation with FDI. The theories backing this perspective mostly stem from export-platform FDI, vertical FDI, cross-border M&A, suggesting that foreign investment activities complement export trade. Therefore, a decrease in trade costs will stimulate enterprises to engage in FDI [35,36]. In recent years, an increasing number of scholars have substantiated this viewpoint through empirical research [37,38]. Will trade costs inhibit enterprises’ willingness to engage in cross-border M&A? This paper conducts theoretical analysis from the perspectives of search costs, communication costs, and others. Primarily, significant differences in institutional environments, limited information flow, and information asymmetry between the investing country and the host country result in higher search costs [39], hindering foreign enterprises from collecting, disseminating, and updating information on the host country’s relevant legal systems, customs, and consumer preferences, thereby inhibiting enterprises from conducting cross-border M&A. Additionally, substantial cultural and customary differences between the investing country and the host country may lead to language barriers, inaccurate information acquisition, causing uncertainties in investment risks, resulting in higher communication and management costs. For digital enterprises, information accessibility, timeliness, and accuracy are crucial. Therefore, when facing higher trade costs such as increased search and communication costs in the host country, foreign enterprises are deterred from engaging in digital cross-border M&A, thus reinforcing the inhibitory effect of GDPR policies on Chinese digital cross-border M&A. Based on this, this paper presents the fourth research hypothesis:

Hypothesis 4: As trade costs escalate, the impact of GDPR on restraining Chinese enterprises from conducting digital cross-border M&A in the EU becomes more pronounced.

## 4. Econometric models, variables and data descriptions

### 4.1. Econometric model construction

In order to effectively identify the impact of the EU’s GDPR policy on Chinese digital cross-border M&A, this paper treats the implementation of GDPR by the EU as a quasi-natural experiment and uses the Difference-in-Differences (DID) method for empirical estimation. The rationale for considering the GDPR as a quasi-natural experiment lies in its role as an exogenous policy shock that came into force in 2018. This enforcement and fixed time point provide ideal conditions for conducting a Difference-in-Differences (DID) analysis. We compare EU countries affected directly by the GDPR (the treatment group) with non-EU countries that are not impacted by this policy (the control group) to observe changes in China’s digital cross-border M&A activities before and after the implementation of the GDPR. This comparison allows us to identify the net policy effect of the GDPR on China’s cross-border M&A activities.Given substantial heterogeneity among countries in terms of economic development, resource endowments, institutional quality, and various other aspects, this study, prior to conducting DID estimation, utilizes a Propensity Score Matching (PSM) model to select control samples with characteristics as similar as possible to the treatment group. Consequently, this study employs the PSM-DID method for estimation, with the specific implementation steps as follows: firstly, estimating the probability of each individual accepting treatment based on covariates, i.e., using a Logit model to estimate propensity scores; secondly, employing kernel matching to select a control group sample set that matches the treatment group; finally, constructing a DID regression model based on the matched data to estimate the average treatment effect. Therefore, this study constructs the following DID model:


$$\begin{array}{c} {\;\;\;\;\;\;\;\;\;\;\;\;\;\;\;\;\;\;\;\;DMA}_{it}=\alpha_{\text{0}}+\alpha_{\text{1}}{EU}_{i}+\alpha_{\text{2}}{Post}_{t}+\alpha_{\text{3}}{EU}_{i}\times {Post}_{t}+\sum_{k}\alpha_{k}{Controls}_{kit}+u_{it}\;\;\;\;\;\;\;\;\; \end{array}$$ (1)

wherein the host countries are represented by i, and years are represented by t. The explanatory variable ${DMA}_{it}$ indicates the quantity of China’s digital cross-border M&A in host country i in year t.$\;{EU}_{i}$ is a dummy variable that takes the value of 1 if country i is a member of the EU, otherwise it takes the value of 0.$\;{Post}_{t}$ is another dummy variable that takes the value of 1 if the year t is 2018 or later, otherwise it takes the value of 0.$\;{Controls}_{kit}$ are control variables, specifically including the host country’s per capita Gross Domestic Product (pGDP), GDP growth rate (Growth), labor force size (Labor), natural resource endowment (Nature), technological resource intensity (Tech), institutional quality (IQ), bilateral geographic distance (Distance), and shared language (Comlan).$\;\alpha_{\text{0}}$ is a constant term, and $u_{it}$ is a random error term.

### 4.2. Data sources and variable settings

#### 4.2.1. Dependent variable (DMA).

Digital Cross-border Mergers and Acquisitions (DMA): Refers to cross-border acquisitions completed by acquiring entities and target companies within the digital economic sector [40]. Regarding the classification of the digital economy sector, since this study focuses on digital cross-border M&As in China, the “Statistical Classification of the Digital Economy and Its Core Industries (2021)” published by the National Bureau of Statistics of China is adopted as the standard for defining the scope of the digital economy. This classification includes five major categories: 01- Digital Product Manufacturing, 02 – Digital Product Services, 03 – Digital Technology Applications, 04 – Digital Element-driven Industries, and 05 – Digitization Efficiency Enhancement. Categories 01–04 constitute the core industries of the digital economy, forming its foundation. This study further subdivides the 01–04 major categories according to the “Statistical Classification of the Digital Economy and Its Core Industries (2021)” and cross-references them with the UK SIC2007 industry classification provided by the Global M&A Transaction Database (Zephyr). Given that the detailed subcategories allow for one-to-one mapping, a total of thirty-two subdivisions across nine major categories within the digital economic sector are identified. These classifications are clearly presented in Table 3.

**Table 3:** Industry classification of the Digital Economy.

Statistical Classification(2021)	UK SIC2007 industry classification	
01 – Digital Product Manufacturing	26 – Manufacture of Computer, Electronic, and Optical Products	261 – Manufacture of Electronic Components and Circuit Boards 262 – Manufacture of Computers and Peripheral Equipment 263 – Manufacture of Communication Equipment 264 – Manufacture of Consumer Electronics 265 – Manufacture of Measuring, Testing, Navigating, and Control Equipment; Watches and Clocks 266 – Manufacture of Irradiation, Electronic Medical, and Electrotherapeutic Equipment 267 – Manufacture of Optical Instruments and Photographic Equipment 268 – Manufacture of Magnetic and Optical Media
	27 – Manufacture of Electrical Equipment	271 – Manufacture of Electric Motors, Generators, Transformers, and Electricity Distribution and Control Apparatus 272 – Manufacture of Batteries and Accumulators 273 – Manufacture of Wiring and Wiring Devices 274 – Manufacture of Electric Lighting Equipment 275 – Manufacture of Domestic Appliances 279 – Manufacture of Other Electrical Equipment
	35 – Electricity, Gas, Steam, and Air Conditioning Supply	351 – Electricity Generation, Transmission, and Distribution 352 – Manufacture of Gas; Distribution of Gaseous Fuels through Mains 353 – Steam and Air Conditioning Supply
02 – Digital Product Services	63 – Information Service Activities	631 – Data Processing, Hosting, and Related Activities; Web Portals 639 – Other Information Service Activities
03 – Digital Technology Application	60 – Programming and Broadcasting Activities	601 – Radio Broadcasting 602 – Television Programming and Broadcasting Activities
	61 – Telecommunications	611 – Wired Telecommunications Activities 612 – Wireless Telecommunications Activities 613 – Satellite Telecommunications Activities 619 – Other Telecommunications Activities
	62 – Computer Programming, Consulting, and Related Activities	620 – Computer Programming, Consulting, and Related Activities
04 – Digital Factor-Driven Industry	58 – Publishing Activities	581 – Publishing of Books, Periodicals, and Other Publishing Activities 582 – Software Publishing
	59 – Film, Video, and TV Program Production, Sound Recording, and Music Publishing Activities	591 – Film, Video, and TV Program Activities 592 – Sound Recording and Music Publishing Activities

The data for DMA is sourced from Zephyr and spans the period from 2009 to 2021. This study specifically extracted all transactional data pertaining to China’s cross-border acquisitions by setting the acquiring country as China, the target country as non-China, and the transaction type as M&A. During the sampling period, a total of 2523 cross-border M&A involving China were identified. Applying the previously mentioned classification method for the digital economic industry, the subset of digital cross-border M&A amounted to 546 cases.

**Fig 1:**
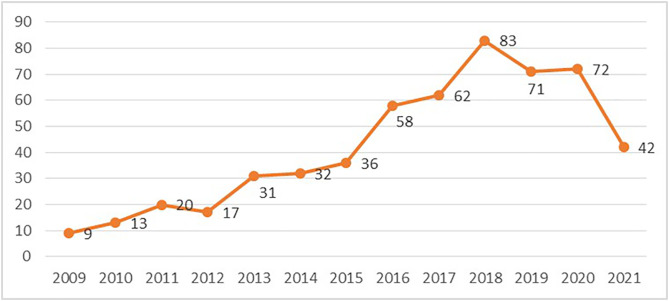
Line graph of China’s digital cross-border M&A.

Fig 1 displays a line graph illustrating the number of completed DMA by China from 2009 to 2021. The figure illustrates a clear upward trend in the number of digital M&As completed before 2018, rising from 9 in 2009–83 in 2018. This growth can likely be attributed to the rapid development of China’s digital economy, increasing market demand, and a heightened willingness to invest internationally. In particular, against the backdrop of a booming internet and e-commerce sector, companies sought to enhance their digital capabilities by engaging in international M&A activities.However, after 2018, this trend changed significantly, with the number of completed digital M&As declining sharply, reaching only 42 in 2021, a level comparable to that of 2015. This shift is closely related to the implementation of the EU’s GDPR in 2018. The GDPR introduced stringent data protection requirements, which led to increased compliance costs and legal risks for many Chinese companies, thereby dampening their M&A activities. To ensure data stability and mitigate heteroscedasticity, this study employed the natural logarithm ln (1 + the number of digital M&A) for the dependent variable DMA.

#### 4.2.2. Moderating variables.

(1) The infrastructure of digitalization (Dinfra). This paper adopts a factor analysis method to construct an indicator system (Dinfra) evaluating the level of digital infrastructure development based on two aspects: the application and the scale of information and communication technologies (ICT). This system delineates six secondary indicators under two primary indicators, enabling a multidimensional assessment of the host country’s digital infrastructure development level, as presented in Table 4. Data for each secondary indicator are sourced from the World Bank’s WDI database.

**Table 4:** Indicator system of digital infrastructure level in the host countries.

Target	Primary indicators	Secondary indicators	Indicator interpretation	Data sources	Unit
Digital infrastructure construction level index system	ICT applications	Fixed broadband penetration	The ratio of fixed broadband subscriptions to the total population of the country	WDI	per 100 people
		Landline telephone penetration	The ratio of landline subscriptions to the total population of the country	WDI	per 100 people
		Mobile phone penetration	The ratio of mobile cellular subscriptions to the total population of the country	WDI	per 100 people
		Internet penetration	The ratio of Internet users to the total population of the country	WDI	%
	ICT scale	Scale of ICT imports	The ratio of ICT merchandise imports to the country’s total merchandise imports	WDI	%
		Scale of ICT exports	The ratio of ICT merchandise exports to the country’s total merchandise exports	WDI	%

(2) Gross Domestic Product (GDP). The size of a nation’s GDP represents its market scale and consumption potential. The data is sourced from the World Bank’s WDI database. To reduce potential heteroskedasticity in the data, this study conducted a natural logarithm transformation on GDP when constructing the model.
(3) Trade Costs (TC). Trade costs refer to all expenses incurred in delivering a product to the final user beyond the marginal cost of producing goods. These include policy barriers, local distribution costs, information expenses, transportation expenses, among others [41]. This study adopts the methodology proposed by Novy [42] to compute the bilateral trade costs between China and the host country. The formula is as follows:

$$\;\;\;\;\;\;\;\;\;\;\;\;\;\;\;\;\;\;\;\;\;\;\;\;\;\;\;\;\;\;\;\;\;\;\;\;\;\;\;\;\;\;\;\;\;\;\;\;{\text{TC}}_{ijt}={\left(\frac{X_{ii}X_{jj}}{X_{ij}X_{ji}}\right)}^{\frac{\text{1}}{\text{2}(\rho -\text{1})}}-\text{1}\;\;\;\;\;\;\;\;\;\;\;\;\;\;\;\;\;\;\;\;\;\;\;\;\;\;\;\;\;\;\;\;\;\;\;\;\;\;\;\;\;\;\;\;\;\;\;\;\;\;\;\;\;\;\;\;\;\;\;\;\;\;\;\;\;\;\;\;\;\;$$ (2)

among them, i and j represent China and the host country.$\;X_{ij\;}$ and ${\;X}_{ji}$ respectively represent the export value of country i to country j and country j to country i. $X_{ii}$ and $X_{jj}$ are the domestic sales of country i and country j respectively. These values indicate the disparity between a country’s overall output and its total foreign exports [43]. The parameter ρ refers to the alternative elasticity of products and has been set at 8. The bilateral trade flows and export data are sourced from the IMF (Direction of Trade Statistics) database, while GDP data is obtained from WDI.

#### 4.2.3. Control variables.

This paper focuses on the following control variables: (1) Per Capita Gross Domestic Product (pGDP) and Gross Domestic Product Growth Rate (Growth). A nation’s pGDP and Growth reflect its economic development and market growth potential. Higher levels of economic development and market potential attract more foreign investments for cross-border M&A [10]. Data for pGDP and Growth are retrieved from the World Bank WDI; (2) Labor Force Scale (Labor). As the labor force in the host country increases, labor costs decrease, thereby attracting foreign investments for cross-border M&A. This study utilizes the total labor force in the host country to assess labor force scale, sourced from the World Bank WDI; (3) Natural Resource Endowment (Nature). Adopting the method by Deng and Yang [44], this study uses the sum of fuel, ore, and metal exports as a percentage of total exports to measure the host country’s natural resource endowment, sourced from the World Bank WDI; (4) Technological Resource Intensity (Tech). The technological resource intensity of the host country significantly impacts foreign enterprises’ cross-border M&A. Following the method by Pan and Jin [45], the proportion of high-tech product exports to total exports is used to indicate a country’s technological resource intensity, sourced from the World Bank WDI; (5) Institutional Quality (IQ). Institutional factors are crucial in determining the location preferences of foreign enterprises for cross-border M&A. This study uses the average of indicators across six dimensions—voice and accountability, political stability, government effectiveness, regulatory quality, rule of law, and control of corruption—to measure the host country’s institutional quality [46], sourced from the World Bank WGI; (6) Bilateral Geographical Distance (Distance). The cost of distance in cross-border M&A can be quantified through geographical distance [47], representing a vital factor in cross-border M&A. This study measures bilateral geographical distance by considering the distance between the two countries’ capitals, sourced from the CEPII database; (7) Common Language (Comlan). Language disparities between nations could escalate communication and information expenses during cross-border M&A, impeding their initiation and completion. Comlan data is sourced from the CEPII database.

To mitigate potential heteroscedasticity in the data, this paper applies natural logarithm transformation to per capita gross domestic product (pGDP), labor force scale (Labor), and bilateral geographical distance (Distance) when constructing the model. Table 5 presents the descriptive statistics of the key variables in the model, comprising total observations, maximum and minimum values, mean, and standard deviation.

**Table 5:** Descriptive statistics for the main variables in the model.

Variables	Obs	Mean	Std. dev.	Min	Max	Data Source
lnDMA	1009	0.2075	0.4875	0.0000	3.1355	Zephyr
EU	1009	0.3221	0.4675	0.0000	1.0000	/
Post	1009	0.3201	0.4667	0.0000	1.0000	/
lnpGDP	995	9.4601	1.3657	5.8251	11.6775	WDI
Growth	998	2.0156	4.3869	−20.4911	24.3704	WDI
lnLabor	943	14.9420	1.7703	10.4985	18.9341	WDI
Nature	815	2.2475	1.5105	−4.4929	4.6047	WDI
Tech	716	14.8965	11.8458	0.0005	69.5995	WDI
IQ	983	0.5064	0.9034	−1.7526	1.8673	WGI
lnDistance	983	8.9786	0.4976	6.8624	9.8677	CEPII
Comlan	983	0.0264	0.1605	0.0000	1.0000	CEPII
Dinfra	690	0.4575	0.1734	0.0000	1.0000	WDI
GDP	955	24.9940	2.3652	18.8303	30.7801	WDI
TC	892	0.2931	0.4340	−1.2400	1.7555	IMF;WDI

## 5. Empirical results and analysis

### 5.1. Benchmark regression results

This study employs Stata 17.0 software to conduct panel data regression analysis for the entire sample in the model. Table 6 reports the changes in the quantity of digital cross-border M&A by China in EU countries before and after the implementation of GDPR. In Column (1), only the core explanatory variables are included, revealing that the coefficient of EU × Post is −0.1090, significant at the 10% level. Column (2) includes core explanatory variables, fixed effects, and control variables (pGDP, Growth, Labor, Nature, Tech, IQ, Dist, and Col). In this case, the estimation coefficient of EU× Post is significantly negative at the 5% level. The baseline regression results demonstrate a significant decrease in China’s digital cross-border M&A in EU countries compared to those in other regions worldwide after the implementation of GDPR. This confirms Hypothesis 1, suggesting that the implementation of GDPR restrains Chinese enterprises from engaging in digital cross-border M&A within the EU.

**Table 6:** Baseline regression estimates results.

	(1)	(2)
EU × Post	−0.1090*	−0.1885**
	(0.0572)	(0.0925)
EU	0.0383	
	(0.0757)	
Post	0.1532***	
	(0.0433)	
Controls	No	Yes
Year fixed effect	No	Yes
State fixed effect	No	Yes
Obs.	1009	646
R-squared	0.0153	0.6315

Note: * p < 0.1, ** p < 0.05, *** p < 0.01, Robust standard errors are clustered at country level in parentheses.

### 5.2. Robustness test

**Fig 2:**
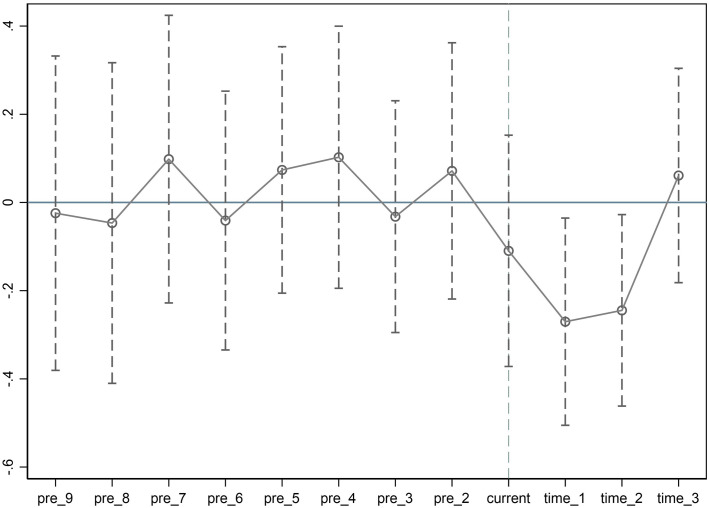
Parallel Trends Test.

(1) Parallel Trends Test. The parallel trends test is a prerequisite for utilizing the DID model, implying that before the implementation of GDPR policy, the trends in digital cross-border M&A between the treatment and control groups should be consistent. Fig 2 presents the parallel trends of Chinese enterprises’ digital cross-border M&A before and after the GDPR policy. The variable ‘current’ represents the virtual year of GDPR policy initiation in 2018. As shown in Fig 2, prior to the implementation of the GDPR, the coefficients for digital cross-border M&As in both the treatment group and the control group are not significant. This indicates that there were no significant differences in trends between the two groups before the GDPR was implemented, thus confirming the robustness of the difference-in-differences (DID) model. This finding suggests that, before the policy intervention, the M&A activities of the treatment and control groups followed similar trends, laying a solid foundation for the subsequent analysis. However, after the implementation of the GDPR, the coefficients for time_1 and time_2 in the parallel trends test are significantly negative, indicating that the GDPR had a negative impact on Chinese digital cross-border M&As post-implementation. The coefficient for time_3 is not significant, suggesting that the effect of the shock was short-lived with limited persistence. These results indicate that the baseline results of this study meet the fundamental assumptions of the DID method, validated through the parallel trends test.

(2) Replacement of Dependent Variable. This study follows Ma et al. 10 by utilizing a binary variable “occurrence of digital cross-border M&A” as the dependent variable for robustness checks. The results in Table 7, Column (1), show that EU × Post is significantly negative at the 10% level, confirming the robustness of the baseline regression results.
(3) Application of Inverse Hyperbolic Sine Transformation. In OLS regression, the natural logarithm transformation of a variable’s value plus 1 has been widely used in the literature but has drawbacks affecting coefficient estimation and standard errors. Drawing from Bellemare and Wichman [48] and Congiu et al. [22], this study applies the inverse hyperbolic sine transformation (Asinh) to the dependent variable – the quantity of digital cross-border M&A – and re-runs Equation (1). The results in Table 7, Column (2), compared to the baseline regression, show an increased absolute value of the EU × Post coefficient, significant at the 5% level, indicating robustness in the findings.
(4) Estimation Methodology Alteration. Since the dependent variable, the count of digital cross-border merger and acquisition (M&A), is a non-negative integer representing count data, the direct application of OLS estimation would likely yield biased results. To address this issue, this study draws on the approach proposed by Cohn et al. [49] and employs Poisson Pseudo-Maximum Likelihood (PPML) estimation for reevaluation. Individual and time fixed effects are controlled to examine the robustness of the conclusions. The results in Table 7, Column (3), indicate that the dependent variable is significantly negative at the 1% level. The main conclusions remain largely unaffected by this change in the estimation model. Furthermore, given the prevalence of zero values in firms’ digital cross-border M&A activities in host countries, a zero-inflated negative binomial (ZINB) model was additionally employed. The results are presented in Column (4) of Table 7. The full ZINB model, which included all control variables, failed to converge due to its high complexity. Nonetheless, the signs of its core coefficients were consistent with our main findings. To obtain reliable estimates, a simplified ZINB model (including only the core variables and fixed effects) was estimated. This model successfully converged, and the coefficient for the core variable remained significantly negative, thereby further corroborating the robustness of our baseline regression results.
(5) Adjustment of Sample Period. Considering the significant impact of the 2019 COVID-19 pandemic on international investments, this section excludes data from the years 2020 and 2021 for a re-run of the regression analysis. The results in Table 7, Column (4), indicate an EU × Post coefficient of −0.2262, significant at the 5% level. Compared to the baseline regression, the absolute value of the coefficient has increased, validating the robustness of the baseline regression results.
(6) Placebo Test. To mitigate endogeneity issues stemming from unobservable characteristics, this study employs both the establishment of pseudo-experimental groups and random simulation methods for placebo testing:

**Table 7:** Robustness test results.

	(1)	(2)	(3)	(4)		(5)	(6)	(7)
EU × Post	−0.1505*	−0.2336**	−0.7395***	−0.6250***	−0.7399***	−0.2262**		
	(0.0765)	(0.1165)	(0.2369)	(0.2323)	(0.2342)	(0.1068)		
nEU × Post							−0.1286	
							(0.0962)	
ukEU × Post								−0.2132**
								(0.0914)
Controls	Yes	Yes	Yes	No	Yes	Yes	Yes	Yes
Year fixed effect	Yes	Yes	Yes	Yes	Yes	Yes	Yes	Yes
State fixed effect	Yes	Yes	Yes	Yes	Yes	Yes	Yes	Yes
Obs.	646	646	447	1009	652	552	646	646
R-squared	0.4536	0.6298				0.6286	0.6275	0.6330

Note: * p < 0.1, ** p < 0.05, *** p < 0.01, Robust standard errors are clustered at country level in parentheses.

① Establishment of Pseudo-Experimental Groups. Initially, to eliminate potential interference from geographically correlated regional shocks on regression outcomes, non-EU European countries within the sample are considered as pseudo-experimental groups for GDPR implementation. These countries include Albania, Belarus, Bosnia and Herzegovina, Switzerland, Georgia, Iceland, Norway, Serbia, Russia, Slovenia, Turkey, and Ukraine, substituted into Equation (1). If the regression coefficient of non-EU European countries is significantly negative, it indicates potential interference from geographically correlated regional shocks. If the coefficient is not significant, it indicates no impact on estimation outcomes. Results in Table 7, Column (5), show the insignificance of the nEU×Post coefficient, indicating no interference from regional shocks, further validating the robustness of the baseline regression results. Furthermore, following Schmitt et al. [50] and Congiu et al. [22], the UK is included as an experimental group within the EU. Despite the official commencement of the Brexit process in March 2017, the EU officially approved Brexit in January 2020, leading to a transition period. During the analysis period of this study until 2020, the UK was part of the EU and continued adhering to GDPR post-exit. Results in Table 7, Column (6), show that the ukEU×Post coefficient is significantly negative at the 5% level, indicating that including the UK as an experimental group does not affect the conclusions of this study.

**Fig 3:**
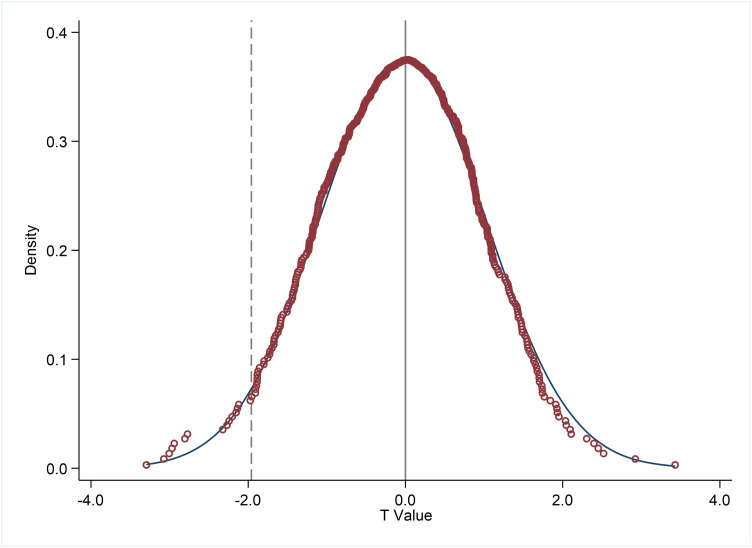
Placebo Test.

② Random Simulation Method. This study randomly assigns the effective date of GDPR to each host country and randomly designates the experimental group for each host country. Equation (1) is then recalculated based on this virtual experimental group. This process is repeated 500 times, and a discrete probability density of virtual regression coefficient t-values, derived from the virtual experimental and control group samples, is plotted in Fig 3. The dotted line in Fig 3 represents the actual regression coefficient t-value. Fig 3 illustrates that the t-values of virtual regression coefficients estimated through the placebo test are distributed around zero, following a normal distribution. In contrast, the actual regression coefficient t-value is clearly an outlier, lying beyond the range of estimates from random simulation, aligning with the expected outcome of the placebo test. This finding enhances the credibility of our research conclusions and further supports the practical impact of the GDPR policy on cross-border M&As.

### 5.3. Heterogeneity analysis

Given disparities in economic development among different nations, the impact of GDPR policy implementation on China’s digital cross-border M&A may exhibit heterogeneous characteristics. This study categorized host countries into high-income and low-to-middle-income groups based on the World Bank’s WDI income level criteria, conducting grouped regressions.

Table 8, Columns (1) and (2), respectively present regression outcomes for the impact of GDPR policy on China’s digital cross-border M&A for the low-to-middle-income country group without and with control variables. Columns (3) and (4) of Table 8 show the corresponding outcomes for the high-income country group without and with control variables. The research reveals that irrespective of the low-to-middle-income or high-income country groups, the estimated coefficient of EU × Post consistently remains significantly negative. Notably, the EU × Post coefficient for the high-income country group is −0.2158, indicating a higher absolute value compared to the low-to-middle-income country group. This suggests that the inhibitory effect of GDPR policy on China’s digital cross-border M&A is more pronounced in high-income countries compared to low-to-middle-income ones. Potential reasons for this phenomenon include: Firstly, income disparities in host countries necessitate varying wage costs for digital foreign enterprises. Investing directly abroad in high-income countries would entail higher wage costs, consequently raising the productivity threshold for export platform and production-type enterprise investments, thereby increasing fixed asset investment costs and diminishing China’s inclination to invest in high-income countries. Secondly, compared to low-to-middle-income countries, high-income countries might have stricter standards for digital firms regarding factory specifications, environmental regulations, and advanced equipment, leading to escalated fixed costs [51], thereby restraining China’s digital cross-border M&A in high-income countries. Thus, the implementation of GDPR policy in high-income countries is likely to curtail the demand for more digital cross-border M&A.

**Table 8:** Heterogeneity test regression results.

	(1)	(2)	(3)	(4)
	**Low to middle income**		**High income**	
EU × Post	−0.1536***	−0.1832**	−0.2005**	−0.2158*
	(0.0390)	(0.0783)	(0.0934)	(0.1140)
Controls	No	Yes	No	Yes
Year fixed effect	Yes	Yes	Yes	Yes
State fixed effect	Yes	Yes	Yes	Yes
Obs.	345	108	653	538
R-squared	0.3833	0.5686	0.6450	0.6365

Note: * p < 0.1, ** p < 0.05, *** p < 0.01, Robust standard errors are clustered at country level in parentheses.

## 6. Mechanism test

To further evaluate the inhibitory effect of GDPR on Chinese companies’ digital cross-border M&A, this study introduces interaction terms between core and moderating variables based on the baseline regression. Model [2] is thus formulated to conduct tests for moderation effects:


$${DMA}_{it}=\alpha_{\text{0}}+\alpha_{\text{1}}{EU}_{i}\times {Post}_{t}+\alpha_{\text{2}}{EU}_{i}\times {Post}_{t}\times M_{it}+\alpha_{\text{3}}M_{it}$$

$$\begin{array}{c} \;\;\;\;\;\;\;\;\;\;\;\;\;\;\;\;\;\;\;\;\;\;\;\;\;\;\;\;\;\;\;\;\;\;\;\;\;\;\;\;\;\;\;\;\;\;\;\;\;\;+\;\sum_{k}\alpha_{k}{Controls}_{kit}+u_{it}\;\;\;\;\;\;\;\;\;\;\;\;\;\;\;\; \end{array}$$ (3)

Wherein $M_{it}$ represents the moderating variable. This study emloys the host country’s digital infrastructure level (Dinfra), gross domestic product (GDP), and trade costs (TC) as moderating variables.

Firstly, this study introduces interaction terms between core variables and digital infrastructure to examine the moderating effect of digital infrastructure level. As shown in Table 9, Column (1), the estimated coefficient for EU × Post × Dinfra is 0.7957, significant at the 10% level. This indicates that the enhancement of the host country’s digital infrastructure construction contributes to alleviating the negative impact of GDPR policies on digital cross-border mergers and acquisitions, validating the hypothesis 2. Secondly, this paper introduces interaction terms between core variables and gross domestic product (GDP) to study the moderating effect of economies of scale. As indicated in Table 9, Column (2), the estimated coefficient of EU × Post × GDP is positively significant at the 1% level. This signifies that the larger the host country’s economy, the weaker the inhibitory effect of GDPR policy on China’s digital cross-border M&A, confirming Hypothesis 3. Lastly, this paper introduces interaction terms between core variables and trade costs to examine the moderating effect of trade costs. As shown in Table 9, Column (3), the estimated coefficient of EU × Post × TC is negatively significant at the 5% level, indicating that the higher the trade costs between the host country and China, the stronger the inhibitory effect of GDPR policy on China’s digital cross-border M&A, confirming Hypothesis 4.

**Table 9:** Moderation effect model regression results.

	(1)	(2)	(3)
	**Dinfra**	**GDP**	**TC**
EU × Post	−0.5633**	−1.8547***	−0.0898
	(0.2657)	(0.6107)	(0.0949)
EU × Post ×Dinfra	0.7957*		
	(0.4661)		
Dinfra	0.0672		
	(0.3803)		
EU × Post ×GDP		0.0635***	
		(0.0233)	
GDP		−0.2549	
		(0.3338)	
EU × Post ×TC			−0.3694**
			(0.1598)
TC			0.4465*
			(0.2379)
Controls	Yes	Yes	Yes
Year fixed effect	Yes	Yes	Yes
State fixed effect	Yes	Yes	Yes
Obs.	565	633	610
R-squared	0.6514	0.6418	0.6566

Note: * p < 0.1, ** p < 0.05, *** p < 0.01, Robust standard errors are clustered at country level in parentheses.

## 7. Conclusion and policy recommendations

### 7.1. Conclusion

With the implementation of the EU’s GDPR, compliance in data matters has garnered significant attention across various sectors. However, GDPR not only sets compliance requirements for digital companies but also exerts substantial influence on the acquisition activities of acquiring parties. This study has constructed a panel dataset of Chinese digital cross-border M&A from 2009 to 2021 by matching data from Zephyr, WDI, CEPII, and IMF databases. Utilizing the PSM-DID method, this research evaluates the policy effects of the EU GDPR implementation on Chinese digital cross-border M&A. The research findings are as follows: (1) The implementation of GDPR significantly negatively impacted Chinese enterprises conducting digital cross-border M&A in the EU. This conclusion remains valid after conducting placebo tests, parallel trend analysis, and various robustness checks. (2) Heterogeneity analysis based on income levels revealed that, in comparison to middle- and low-income countries, GDPR policies in high-income countries had a more pronounced inhibitory effect on Chinese digital cross-border M&A. (3) Analysis of moderating effects indicated that the improvement of the digital infrastructure level, the expansion of EU market size and reduced trade costs weaken GDPR’s inhibitory effect on Chinese enterprises engaging in digital cross-border M&A in the EU.

### 7.2. Policy recommendations

This paper implies several policy insights, including the following aspects: (1) Providing comprehensive data legislation information: The Chinese government should establish and publish data compliance guidelines for major global markets (e.g., the EU, US) to help companies better understand international data regulations and reduce compliance risks in cross-border M&A. (2) Offering professional legal support: The government should offer legal aid to Chinese enterprises facing data compliance investigations or penalties abroad, especially in cases of data compliance reviews or sanctions, to ensure their legal rights are protected. (3) Building a robust data regulatory framework: While the GDPR requires companies to appoint Data Protection Officers (DPOs) for compliance, external regulatory oversight is equally crucial. Governments should establish standardized certification for DPOs and continuously monitor their performance to ensure compliance with international data regulations. (4) Strengthening Personal Data Protection Measures: Policymakers should enhance the security of personal data storage and transmission. This includes integrating cutting-edge technologies such as artificial intelligence and blockchain into risk assessment, monitoring, early warning, and auditing processes related to the cross-border flow of personal data [52]. These measures are necessary to meet the stringent requirements of the GDPR regarding data privacy and to reduce the risk of data breaches in cross-border M&A. (5) Unifying cross-border data flow rules: Regulators should strengthen coordination between countries and regions on data protection laws by promoting international cooperation to develop consistent, transparent standards for cross-border data flows. This would help reduce the compliance burden for companies operating across multiple jurisdictions.

### 7.3. Discussion

Although this study provides new insights into the impact of the GDPR on Chinese digital cross-border M&A activities, several limitations remain that should be addressed in future research. First, the data used in this study only covers M&A transactions by Chinese digital firms, without fully incorporating data from other countries and regions. This may limit the ability to capture the global impact of the GDPR on digital cross-border M&As. Future research should focus on digital cross-border M&As on a global scale to enhance the general applicability of the findings. Second, due to data limitations and the complexity of measuring data protection regimes, this study does not provide a detailed comparison of data protection systems in different regions or jurisdictions (such as the U.S. and China). Future studies could consider developing indicators to measure the level of data protection across different countries and regions, enabling cross-national comparative analyses for a more comprehensive understanding of the impact of data protection laws on digital cross-border M&As. Finally, due to the large number of countries involved in this study, obtaining comprehensive data on key and control variables at the firm level proved challenging. Moreover, the focus of this study differs from firm-level analyses. Therefore, this paper primarily analyzes the policy effects of the GDPR at the macro (national) level, and does not further explore industry- or firm-specific impacts. Future research could use case studies to explore how data protection laws influence transaction strategies, valuations, due diligence processes, and post-merger integrations, providing a deeper understanding of the complex relationship between policy and M&A activities. By overcoming these limitations, future research will contribute to a better understanding of the long-term effects of data protection laws on the global digital economy and offer more practical policy recommendations for cross-border M&A activities.
